# The Characteristic and Distribution of Shale Micro-Brittleness Based on Nanoindentation

**DOI:** 10.3390/ma15207143

**Published:** 2022-10-13

**Authors:** Liu Yang, Yuting Mao, Duo Yang, Zhenchuan Han, Sheng Li, Jianchao Cai, Manchao He

**Affiliations:** 1 State Key Laboratory for GeoMechanics and Deep Underground Engineering, China University of Mining and Technology, Beijing 100083, China; 2 School of Mechanics and Civil Engineering, China University of Mining and Technology, Beijing 100083, China; 3 Research Institute of Exploration and Development, Xinjiang Oilfield Company, PetroChina, Karamay 834000, China; 4 State Key Laboratory of Petroleum Resources and Prospecting, China University of Petroleum, Beijing 102249, China

**Keywords:** shale, nanoindentation, brittleness index, friability

## Abstract

Shale is a special kind of rock mass and it is particularly important to evaluate its brittleness for the extraction of gas and oil from nanoporous shale. The current brittleness studies are mostly macro-evaluation methods, and there is a lack of a micro-brittleness index that is based on nanoindentation tests. In this paper, nanoindentation tests are carried out on the surface of shale to obtain mechanical property, and then a novel micro-brittleness index is proposed. Drawing a heat map by meshing indentation, the distribution characteristics of the brittleness index for the surface of shale and the variation laws between the mineral and brittleness index are explored. The results showed that the dimensionless brittleness index involved parameters including indentation irreversible deformation, elastic modulus, hardness and fracture toughness. The micro-brittleness index of the shale ranged from 7.46 to 65.69, and the average brittleness index was 25.837. The brittleness index exhibited an obvious bimodal distribution and there was great heterogeneity on the surface of shale. The crack propagation channels were formed by connecting many indentation points on the shale surface with high brittleness. The total brittleness index of quartz minerals was high, but the cementation effect with different minerals was various. Although the general brittleness of clay was low, the high brittleness index phenomenon was also exhibited. Studying the micro-brittleness of shale provides a more detailed evaluation for the shale friability, which is used to determine the optimal shale oil and gas recovery regime.

## 1. Introduction

With the development of advanced hydraulic fracturing and horizontal drilling technologies, the exploration and development of shale gas reservoirs has achieved great success. Shale is a special rock material including multi-component minerals, widely developed pores, fractures and bedding, which is liable to break and muddy [1,2]. In previous studies, a complete shale core was required to obtain macroscopic mechanical parameters to study the evaluation of shale brittleness. However, due to the fragility of deep shale in the coring process, the existing brittleness evaluation methods for shale have many shortcomings [3]. In recent years, many advances have been made in nanoscale microscopic damage indentation experiments to measure micro-mechanical parameters. Compared with conventional macroscopic experiments, the tested samples in nanoindentation experiments are smaller and easier to obtain, which gives much convenience in mechanical experiments [4,5,6].

The brittleness of shale is a key factor that determines the failure characteristics of shale under cyclic loading. By concluding all brittleness index evaluation methods in previous research, four kinds of typical evaluation methods can be obtained. The first is related to the mechanical strength parameter, i.e., the uniaxial compressive strength and tensile strength are referred to give the ratio of both. The higher the ratio, the more brittle the rock [7,8,9,10]. The second is based on the stress–strain curve of the rock [11,12,13,14]. The third method uses elastic parameters such as elastic modulus and Poisson’s ratio to evaluate rock brittleness. The development of this method based on elastic constants generally assumes that a greater elastic modulus and a smaller Poisson’s ratio favor fracturing, hence indicating a higher brittleness of the rock [15,16,17]. In the fourth method, the mineral composition evaluation method is introduced, and the ratio of the weight and volume fraction of the component minerals that are beneficial to brittle failure to the component minerals is used as the brittleness index in the hydraulic fracturing of shale gas reservoirs [18,19,20]. In addition to the above, other evaluation methods such as abnormal logging data and changes in the prominence of the internal friction angle are also used to evaluate shale brittleness [21,22].

Nanoindentation tests are popular in evaluating the local mechanical property of material and are widely used in analyzing the local mechanical characteristics of geomaterial or rock materials [23,24,25]. The influence of total organic carbon (TOC) and clay on the mechanical properties of shale are explored by conducting nanoindentation tests, and it can be found that with an increase in the TOC and clay, the elastic modulus decreases gradually. The increasing thermal maturity of the TOC will induce the rising of the elastic modulus [26]. The increasing TOC will result in the strength of the anisotropy of the shale [27]. For the study of the variations among elastic modulus *E*, hardness *H* and fracture toughness *K_c_*, it can be found that an increasing tendency is exhibited between *K_c_* and *E*, and the linear variation is obtained between *E* and *H* [28]. In addition to the above studies, the creep behaviors of shale and the effect of temperature and supercritical CO_2_ on the mechanical properties of shale are investigated [29,30].

However, most of the current research focuses on the brittleness evaluation for the macro-parameters of shale, and the brittleness evaluation for the micro-parameters of shale is rare by using nanoindentation method. This paper takes the shale of the second member of the Paleogene Kongdian Formation in the Cangdong Sag, Bohai Bay Basin as the research object. In this study, the micro-mechanical properties of shale are investigated by conducting nanoindentation tests, and a reasonable micro-brittleness index is proposed. Through a series of scanning electron microscope (SEM) and quantitative mineral evaluation tests, the changes in the brittleness index and mineral composition distribution were analyzed, and the distribution changes in the shale surface brittleness index were further analyzed.

## 2. Methodology

### 2.1. Geological

**Figure 1:**
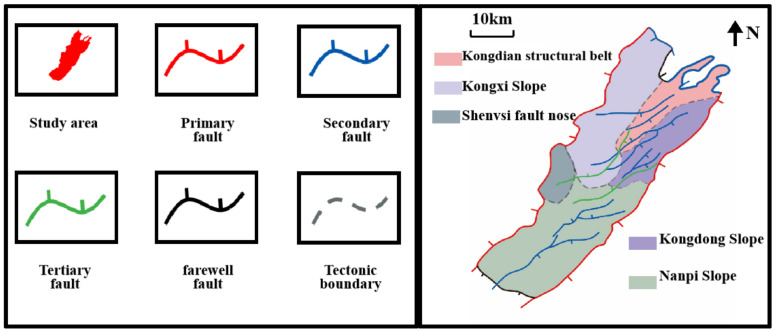
Geographical location and geological structure of Cangdong Sag.

The Bohai Bay Basin is a petroliferous basin in eastern China. In recent years, the exploration of shale oil in the Cangdong Sag of the Bohai Bay Basin (Figure 1) has achieved tremendous breakthrough [31]. The Cangdong Sag is located in the middle of the Bohai Bay Basin, between the Cangdong and Xuxi faults in both the east and west direction, with the Kongdian and Dongguang bulges retaining the Sag in the south–north direction. Additionally, it is a secondary structure unit in the Huanghua depression of the Bohai Bay Basin, with an area of about 1800 km^2^. During the sedimentation of the Second Member of Kongdian Formation (EK_2_), the lake basin of the Cangdong Sag covered a large area. Controlled by the EK_2_ trough, an oil-bearing system with EK_2_ as the source rock and multiple sets of Kongdian Formation as reservoirs has formed.

### 2.2. Sample

**Figure 2:**
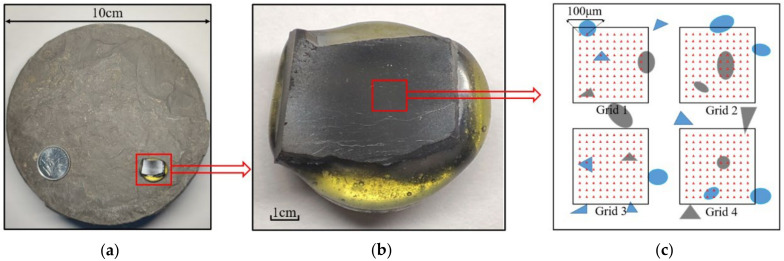
Schematic diagram of sample acquisition: (**a**) full diameter core, (**b**) shale rock sample for nanoindentation, (**c**) schematic diagram of grid indentation.

The sample in this paper was taken from the shale in the middle ring zone in the trough area. The shale in this area is mainly composed of quartz, carbonate minerals and clay minerals. The tested shale sample was mixed, curing with an epoxy resin and a curing agent whose size was set as a length of 5 mm, width of 5 mm and thickness of 3 mm. Then, the cured samples were polished by using the polished section with 15 μm, 9 μm, 3 μm and 0.5 μm to ensure the smoothness of the sample, as displayed in Figure 2. Finally, the sample was placed on the sample table of the multifunctional ion thinning instrument for argon ion polishing. To obtain the mineral component of the shale sample, the shale sample in the same position was extracted to make experimental powder, which was performed by X-ray diffraction (XRD).

### 2.3. Experimental Method

#### 2.3.1. X-ray Diffraction (XRD)

The crushed samples were mixed with ethanol, hand ground and then smear mounted on glass slides for XRD. The experiments were conducted on a LECO C-S diffractometer (Leco Company, St. Joseph, MI, USA) using Co Kα-radiation produced at 45 kV and 35 mA. The diffracted beam was measured with a scintillation detector with a counting time of 20 s for each step of 0.02° 2θ. Diffract grams were recorded from 2° to 76° 2θ. By further analyzing the crystalline content of the shale, it had a specific X-ray diffraction pattern, and the content of various minerals inside the sample could be obtained by analyzing the peak intensity of the X-ray diffraction pattern.

#### 2.3.2. Mineral Petrological Detection

QEMSCAN is the abbreviation of a comprehensive automatic mineral petrological detection method and the full name is quantitative evaluation of minerals by SCANning electron microscopy. QEMSCAN became a registered trademark of the FEI in 2009. A quantitative mineralogical analysis was provided by the QEMSCAN evaluation system (FEI Company, Hillsboro, OR, USA). This scan evaluation was characterized by Quanta 650F and energy-dispersive X-ray spectroscopy (EDS). This combination performs a mineralogical analysis of the sample by scanning the surface of the sample. QEMSCAN determines the mineralogical composition of the test sample by scanning the surface of the epoxy block in raster mode (one pixel at a time) as it scans the minerals. The specific steps are: First, the elemental characteristics of the pixels are determined using an EDS spectrometer (Nippon PIGAKV Motor Co., Ltd., Tokyo, Japan) and are compared with a proprietary database of known minerals to determine the mineral characteristics. The analysis of one pixel takes about two to four milliseconds, and over a million pixels can be mapped per hour. The resolution of the analysis can vary between 1 μm and 2 mm. After the mineralogy of each pixel is determined, all pixel information is combined to generate an image for quantitative mineralogy analysis.

#### 2.3.3. SEM

The SEM test was performed with a Japanese JEOL JSM-7800F field emission scanning electron microscope (Nippon PIGAKV Motor Co., Ltd., Tokyo, Japan), which consists of a vacuum system, an electron optical system and an imaging system. The electron optical system captures the scanning electron beam and serves as the excitation source for physical signal generation. The minimum resolution is 3 nm, and the magnification is 4~100,000 times. In addition, the energetic incident electrons bombarding the surface of the material are emitted from the electron optical system, and the back-reflected electrons and diffraction of the X-rays generated by this process are received and imaged by the probe.

### 2.4. Nanoindentation Technique

#### 2.4.1. Principle

**Figure 3:**
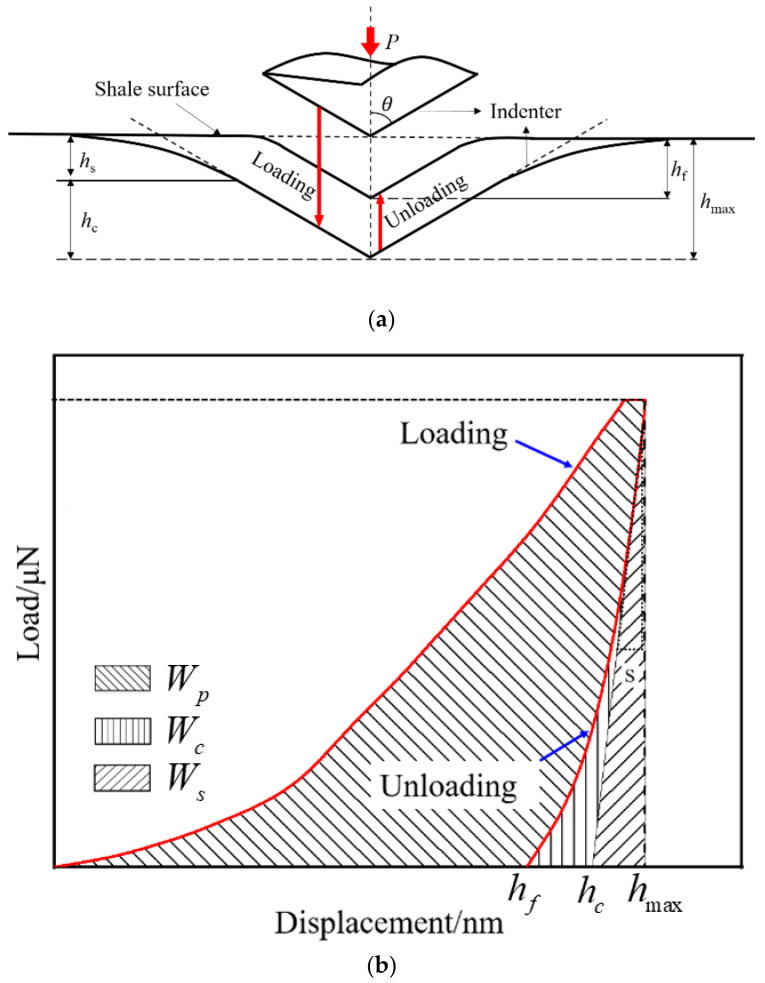
Nanoindentation experiment process: (**a**) schematic diagram of loading and unloading, (**b**) schematic illustration of indentation load-displacement curve. *P* is the loading stress; S is the contact stiffness; *W_p_* is plastic energy; *Ws* is elastic energy; *W_c_* is *D*-value between plastic energy and pure plastic energy.

During the nanoindentation test, the variations between load and displacement can be obtained by controlling the loading head load sample with the high-precision sensor. As shown in Figure 3a, nanoindentation includes three stages, i.e., the loading, keep loading and unloading stage. Elastoplastic deformation appears in the loading stage, and the loading increases with an increase in pressed depth. The influence of the sudden change in loading on mechanical parameters can be removed in the keep loading stage. In the unloading stage, with the decreasing loading, the pressed depth will have part recovery, which is assumed as elastic deformation and is used to calculate the mechanical property of the pressed point.

#### 2.4.2. Experimental Program

To ensure the most valuable indentation size, the initial indentation test was performed before conducting a nanoindentation test of shale. As shown in Figure 3b, in order to analyze the mechanical properties of a single mineral, the maximum indentation loading, loading/unloading rate and holding time in this text were set as 500 μN, 100 μN/s and 5 s, respectively. Due to the strong heterogeneity of the sample surface, a large number of indentation points were applied with the grid indentation method, which makes it easy to conduct a statistical analysis on the micro-mechanical properties of the samples. As shown in Figure 2c, four indentation grids were set for comparative analysis. In each indentation grid, the distance between the indentation points was 100 μm, and each indentation grid contained 10 × 10 indentation points.

#### 2.4.3. Calculation Method of Brittleness Index

The displacement–loading curve corresponds to each indentation point. As shown in Figure 3b, *h*_max_ represents the maximum indentation depth, *h_f_* is the indentation depth after absolute unloading, *h_s_* and *h_c_* are the unrecoverable deformation depths at the edge of the indentation and at the center of the indentation, respectively.

The Oliver and Pharr method was introduced to calculate the *H* and *E* of shale [32].

The *H* can be obtained by:

(1) $$H=\frac{P_{\max}}{A_{c}}$$

where *P*_max_ is the maximum loading, N; and *A_c_* is contacting area, m^2^. For the ideal Bose indenter, it can be obtained by:

(2) $$A_{c}=24.5h_{c}{}^{2}$$

*E* can be calculated by:

(3) $$E_{r}=\frac{\sqrt{\pi }S}{2\beta \sqrt{A_{c}}}$$

where *S* is the contact stiffness that is the slope of the initial unloading stage, *β* is the parameter related to the shape of the indenter and the *β* of the ideal indenter is 1.034.

*K_c_* is calculated by the energy method [33]:

(4) $$K_{c}=\sqrt{G_{c}E_{r}}$$

where *E_r_* is the modified elastic modulus, GPa; and *G_c_* is the critical energy release rate.

In the calculation of *G_c_*, the total energy in the indentation deformation is composed of elastic energy and plastic energy, and the plastic energy consists of pure plastic energy and the energy induced by fracture.

(5) $$W=W_{e}+W_{p}=W_{e}+W_{pp}+W_{f}$$

where *W_f_* represents the energy used to result in a fracture in the indentation deformation, and *W_pp_* is pure plastic energy. Based on above research, it can be obtained (Cheng et al. 2002):

(6) $$\frac{W_{pp}}{W}=1-[\frac{1-3{\left(h_{f}/h_{\max}\right)}^{2}+2{\left(h_{f}/h_{\max}\right)}^{2}}{1-{\left(h_{f}/h_{\max}\right)}^{2}}]$$

Then,

(7) $$G_{c}=\frac{\partial W_{f}}{\partial A_{c}}=\frac{W_{f}}{A_{c}}$$

In the fracture mechanic, the *K_c_* can be determined by the length of the crack that resulted from residual indentation under set loading, which is shown as follows (Lee et al. 2012):

(8) $$K_{c}=\frac{P_{\max}}{l^{3/2}}\prod \left(\frac{E}{H},v,\phi ,\frac{l}{h_{c}}\right)$$

where *l* is the length of the crack, *v* is Poisson’s ratio and ϕ is the shape parameter of the indenter:

(9) $$\frac{K_{c}l^{3/2}}{P_{\max}}=\prod \left(\frac{E}{H},v,\phi ,\frac{l}{h_{c}}\right)$$

Substituting Equations (1) and (2) to Equation (9) yields:

(10) $$\frac{K_{c}l^{3/2}}{H\cdot A_{c}}=\frac{K_{c}l^{3/2}}{H\cdot 24.5h_{c}^{2}}=\prod \left(\frac{E}{H},v,\phi ,\frac{l}{h_{c}}\right)$$

(11) $$\frac{K_{c}}{H\cdot l^{1/2}}=\prod \left(\frac{E}{H},v,\phi ,\frac{h_{c}}{l}\right)$$

(12) $$\frac{K_{c}^{2}}{H^{2}\cdot l}\cdot \frac{H}{E}=\frac{K_{c}^{2}}{HEl}=\prod \left(v,\phi ,\frac{h_{c}}{l}\right)$$

Then, the *B*_1_ can be regarded as:

(13) $$B_{1}=\frac{HEl}{K_{c}^{2}}$$

In the nanoindentation test, the length of the crack can be tested with a high precision electronic scanner, and the grid indentation results in a great deal of experimental work. Hence, the length of crack *l* is replaced by *h_s_*, which is the test data directly from the indentation experiment:

(14) $$h_{s}=\varepsilon \frac{P_{\max}}{S}$$

The horizontal projection of the deformation caused by the indentation at the edge is the crack length *l*. Due to the geometric similarity between *h_s_* and *l*, it can be obtained:

(15) $$h_{s}\propto l$$

So, the brittleness index *B* is defined by:

(16) $$B=\frac{HEh_{s}}{K_{c}^{2}}$$

Substituting Equations (1), (4) and (7) to Equation (16) yields:

(17) $$B=\frac{EHh_{s}}{K_{c}{}^{2}}=\frac{Eh_{s}P_{\max}/A_{c}}{EW_{f}/A_{c}}=\frac{P_{\max}h_{s}}{W_{f}}=2\frac{W_{s}}{W_{f}}$$

where *W_s_* is the elastic recoverable energy of the contacting area of the indenter. The *W_s_*/*W_f_* represents the size of the friability of the indentation point, which further proves the applicability of *B*.

## 3. Results

### 3.1. Mineralogy Information

In order to obtain the mineral component of the shale sample, the XRD tests were performed on the fragments within the experimental shale sample. As shown in Table 1, the mineral component of shale was composed of quartz, potash feldspar, plagioclase, calcite, dolomite, clay minerals, pyrite and illite.

It should be noted that the proportion of carbonate minerals was 49.1% and accounted for the largest part. There are two traditional shale brittleness assessment methods based on mineral composition. The carbonate mineral was regarded as the brittleness mineral and the carbonate mineral was eliminated from the evaluation of brittleness. In this test, the micro-mechanical property was directly used to evaluate the brittleness of the shale.

### 3.2. Mineral Petrological Detection

**Figure 4:**
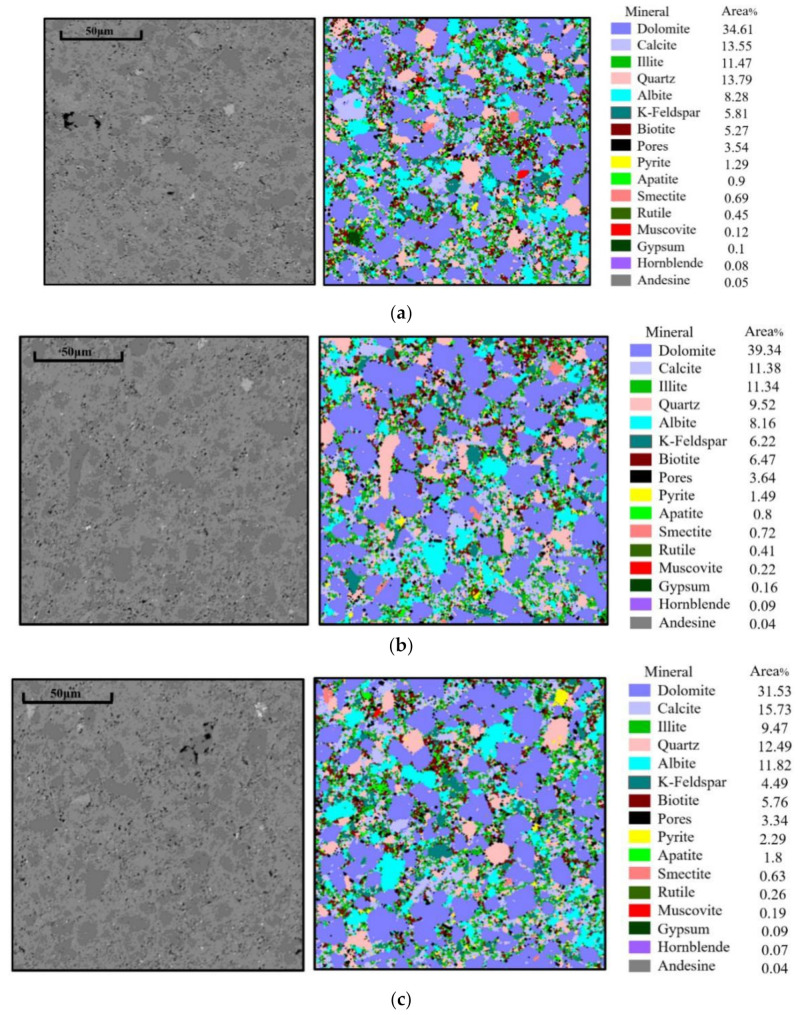

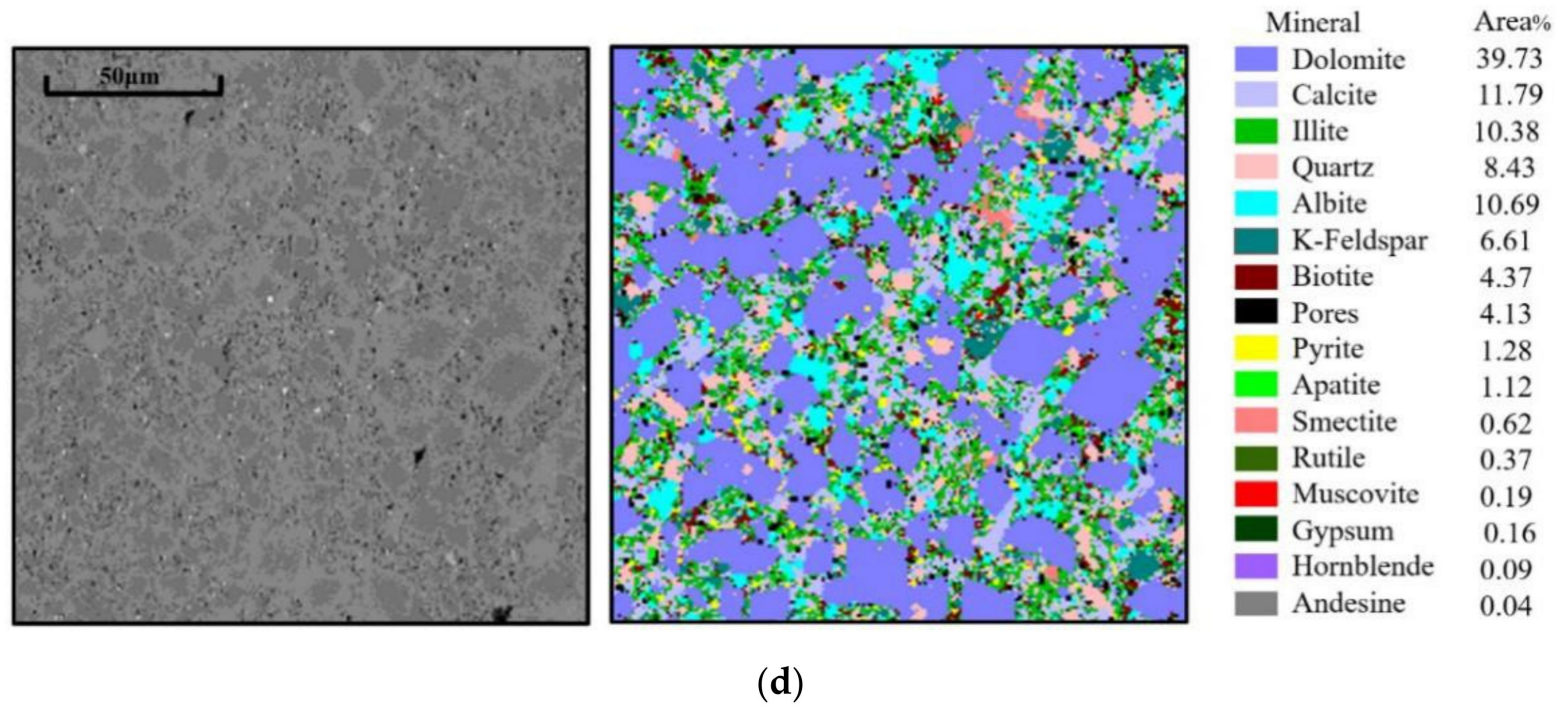
Mineral distribution under QEMSCAN: (**a**) nanoindentation grid 1, (**b**) nanoindentation grid 2, (**c**) nanoindentation grid 3, (**d**) nanoindentation grid 4.

As shown in Figure 4, the surface of shale mostly consisted of dolomite particles, scattered quartz particles and feldspar. The grains were filled with clay containing illite, chlorite and a small amount of biotite, which corresponded to the results of the XRD tests. In grid 1, the quartz, feldspar, carbonate rock mineral and clay mineral content were 13.79%, 14.09%, 48.16% and 17.43%, respectively. In grid 2, the quartz, feldspar, carbonate rock mineral and clay mineral content were 9.52%, 14.38%, 50.72% and 18.53%, respectively. In grid 3, the quartz, feldspar, carbonate rock mineral and clay mineral content were 12.49%, 16.31%, 47.26% and 17.43%, respectively. In grid 4, the quartz, feldspar, carbonate rock mineral and clay mineral content were 8.43%, 17.3%, 51.52% and 15.37%, respectively. Among the four grids, the quartz content in grid 1 was the largest, which was 5.36% higher than that in grid 4. Grid 4 had the highest feldspar content, which was 3.21% higher than grid 1 with the lowest feldspar content. The carbonate mineral content was the highest in grid 4, which was 4.26% higher than that in grid 3. The highest clay mineral content in grid 2 was 3.16% higher than that in grid 4. The difference in mineral content in the four grids was less than 6%, but the distribution of minerals was different, which resulted in the variations in the micro-properties of the shale.

### 3.3. SEM

**Figure 5:**
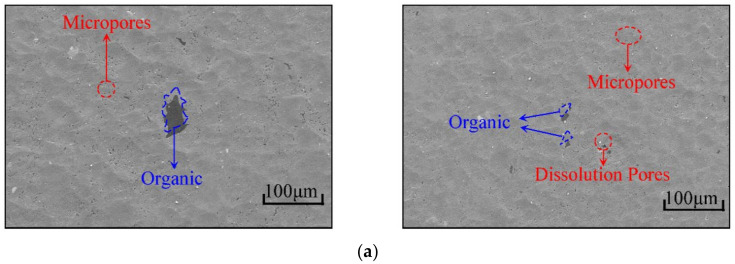

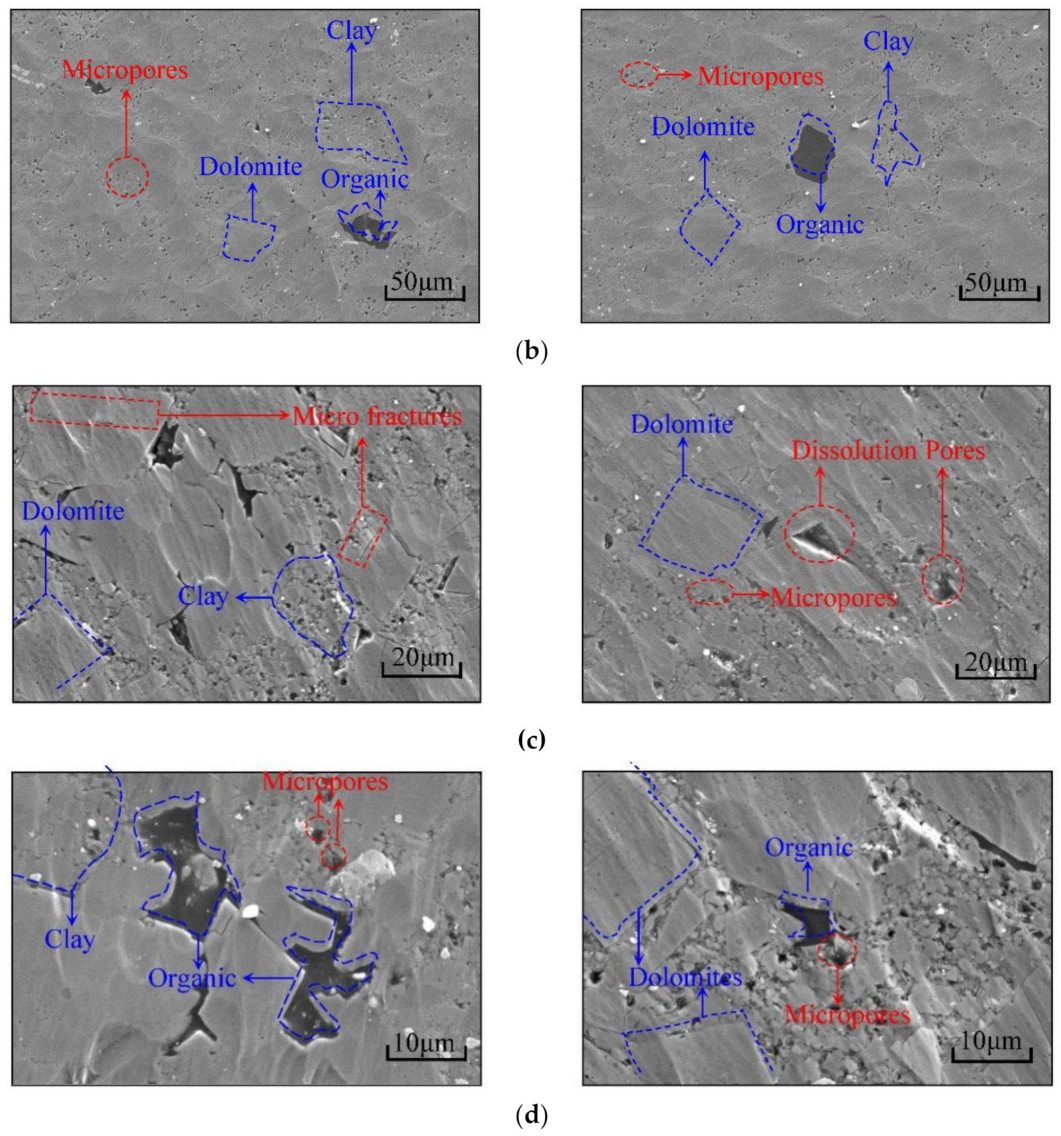
SEM images in different fields of shale: (**a**) 1200× magnification, (**b**) 2000× magnification, (**c**) 5000× magnification, (**d**) 8000× magnification.

Figure 5 shows the SEM images of the shale samples under different horizons, with magnifications of 1200×, 2000×, 5000× and 8000× times, respectively. At 1200 magnification, the mineral particles were scattered on the surface of the shale, along with sporadic organic matter. In addition, the obvious micro-pores and pores formed after mineral exfoliation could be seen. When the magnification was 2000× times, the mineral contour was clear, and the clay minerals filled in the middle of the mineral particles could be observed clearly. The micro-pores in the clay minerals were more obvious, making the clay minerals honeycomb. When the magnification was 5000× times, the mineral shape was clearer. Combined with the mineral distribution obtained by QEMSCAN, the square dolomite particles could be clearly identified, but quartz, feldspar and other particles could not be identified in this mode. In addition to the initial pores on the surface of the shale, there were also many pores caused by the whole or partial exfoliation of massive minerals. Beyond that, there were also microscopic fissures developed at the edges of the mineral grains and pores throughout the mineral grains. At 8000 magnifications, the bonds between minerals could be seen more clearly. In the middle of the lumpy mineral grains, clay minerals and organic matter filled them.

### 3.4. Analysis of Load–Displacement Curves

**Figure 6:**
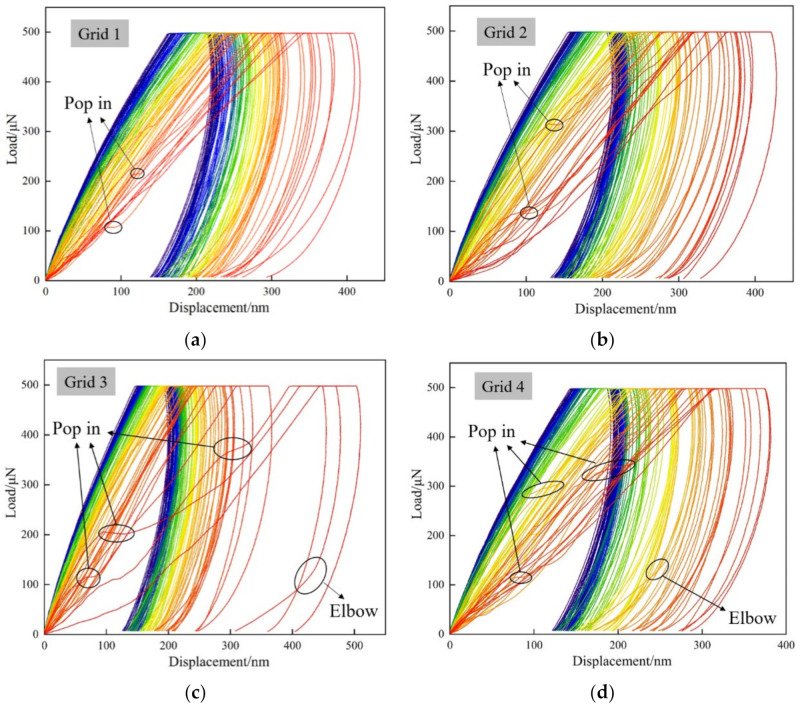
Load–displacement curve: (**a**) nanoindentation grid 1, (**b**) nanoindentation grid 2, (**c**) nanoindentation grid 3, (**d**) nanoindentation grid 4. Blue represents high elasticity and red represents low elasticity.

Each indentation point corresponds to a loading–displacement curve, which represents the mechanical behavior of the mineral composition at the indentation point. Under nanoindentation loading, the displacement loading curve presented a similar shape. As shown in Figure 6, the displacement increased gradually with the increasing load in the loading stage. During the keep loading stage, the displacement continued to increase. The elastic deformation recovered and left residual deformation *h_f_* in the unloading stage. Under the same loading rate and peak load, the trends of the displacement load curves were roughly similar. However, the curve was slightly different due to diversity minerals, and the mechanical parameters were obtained through the analysis of the curve. Among the four grids, the maximum residual indentation depths were 260 nm, 328 nm, 325 nm and 286 nm, respectively, and the minimum residual indentation depths were 50 nm, 52 nm, 45 nm and 48 nm, respectively. It can be seen that *h*_max_ varied greatly, which owed to the different mineral compositions and shale micro-structures. The obvious “pop in” and “elbow” phenomena can be seen in curves, and the phenomenon of “pop in” was relatively common. Such a sudden increase in displacement may have resulted from the micro- and nanoscale pores. These may have been original or may have been micro-cracks produced by the indenter in pressing. “Pop in” mostly occurred at the indentation points with a large indentation depth, and the indentation points were mostly clay minerals. It can be seen from the SEM in Section 3.3 that there were many micro-pores developed in the clay minerals. When the indenter met these pores in the process of pressing, displacement mutation occurred. The elbow phenomenon occurred during the unloading process and was characterized by abrupt changes in the unloading slope. The occurrence of this phenomenon may have been the result of the phase transitions that occurred in the slow lifting of the indenter. This phenomenon can be explained by SEM in Section 3.3, and on the plane, the clay minerals were used as substrates to fill between the large grains. However, in the longitudinal direction, when the indenters experienced a transition from matrix to brittle minerals in the unloading process, the “elbow” phenomenon occurred. It is worth noting that the indentation size was smaller than the grain size of the mineral, and the “elbow” phenomenon mostly appeared in grids 3 and 4.

### 3.5. Mechanical Properties

The micro-mechanical properties of the shale were obtained by a series of nanoindentation experiments, i.e., *E*, *H* and *K_c_*. To better analyze the relationships among *E*, *H* and *K_c_*, the analysis of the normal distribution on these were conducted by Equation (18).

(18) $$y_{E}=\frac{A}{\sqrt{2\pi }\sigma }\exp\left[-\frac{1}{2}{\left(\frac{x-\mu }{\sigma }\right)}^{2}\right]$$

where *σ* is standard deviation and represents the amplitude of the distribution of data and *μ* is the mathematical expectation and characterizes the position of the normal distribution of the data.

#### 3.5.1. Elastic Modulus

**Figure 7:**
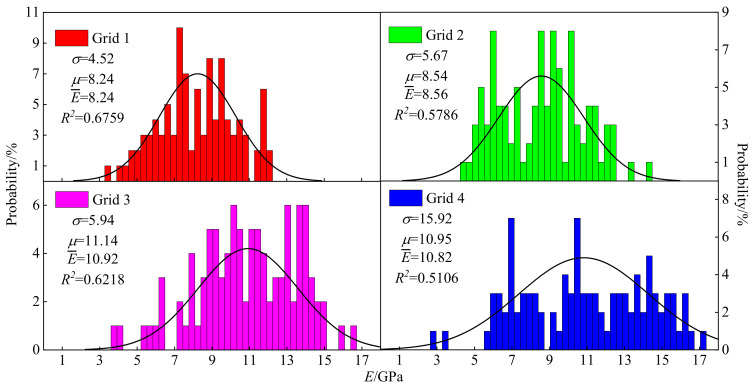
Frequency distribution histogram of elastic modulus. The closer the value of R² is to 1, the better the fit of the regression line to the observed values; on the contrary, the smaller the value of R², the worse the fit of the regression line to the observed values.

The frequency distribution of *E* is displayed in Figure 7 and the fitting correlation coefficients of grid 1, 2, 3 and 4 were 0.6759, 0.5786, 0.6218 and 0.5106, respectively. The varying ranges of *E* of grid 1, 2, 3 and 4 were 3.58~12.07 GPa, 4.45~14.24 GPa, 3.82~16.48 GPa and 2.74~17.16 GPa, respectively. The mean elastic modulus $\bar{E}$ of the four grids were 8.24, 8.54, 10.92 and 10.82 GPa, respectively, and the corresponding mathematical expectations were 8.24, 8.54, 11.14 and 10.95, respectively. It can be seen that the indenters varying from 7 to 10 GPa of *E* accounted for 52% in grid 1 and that $\bar{E}$ was the greatest in grid 3. However, the variance of grid 4 was much higher than that of grid 3, which can be accounted for with the discrete data in grid 4.

#### 3.5.2. Hardness

**Figure 8:**
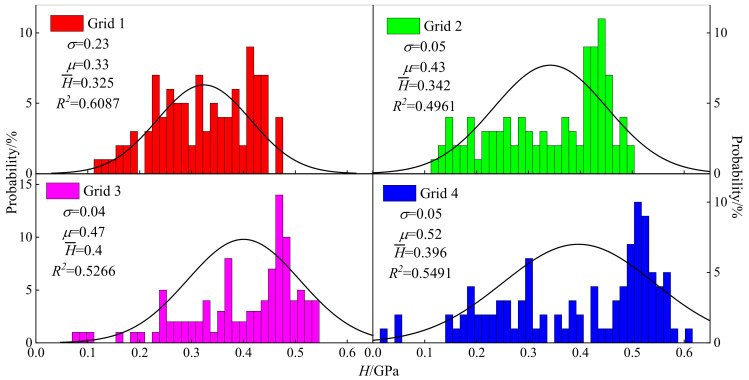
Frequency distribution histogram of *H*. The closer the value of R² is to 1, the better the fit of the regression line to the observed values; on the contrary, the smaller the value of R², the worse the fit of the regression line to the observed values.

The frequency distribution of *H* is displayed in Figure 8 and the fitting correlation coefficients of grid 1, 2, 3 and 4 were 0.6087, 0.4961, 0.5266 and 0.5491, respectively. Except for grid 1, the other distributions in grid 2, 3 and 4 had an obvious negative skew distribution, and the concentration areas of *H* were significant with range of 0.1 GPa. The varying areas of *H* of grid 1, 2, 3 and 4 were 0.122~0.476 GPa, 0.115~0.496 GPa, 0.081~0.546 GPa and 0.146~0.604 GPa, respectively. The average *H* of grid 1, 2, 3 and 4 were 0.325, 0.342, 0.4 and 0.396 GPa, respectively. The concentration ranges of grid 1, 2, 3 and 4 were 0.4~0.5 GPa, 0.4~0.5 GPa, 0.45~0.55 GPa and 0.45~0.55 GPa, respectively, and the proportions of the indenters were 27%, 48%, 46% and 40%, respectively. Grid 4 had the largest hardness distribution interval and the largest average hardness. The mathematical expectations of grid 1, 2, 3 and 4 were 0.33, 0.43, 0.47 and 0.52, respectively, which were higher than $\bar{H}$ owing to the high hardness minerals in the clay.

#### 3.5.3. Fracture Toughness

**Figure 9:**
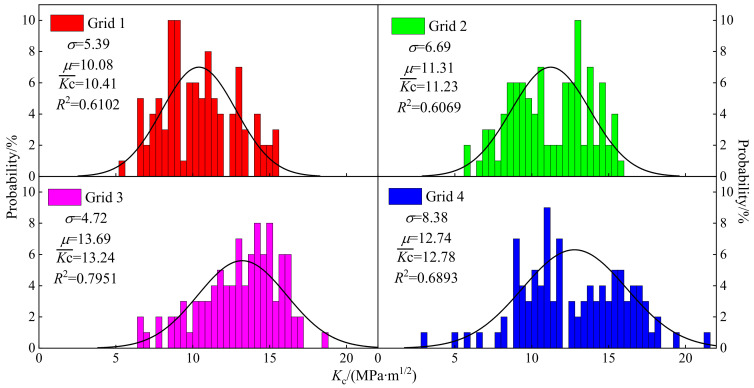
Frequency distribution histogram of *K_c_*. The closer the value of R² is to 1, the better the fit of the regression line to the observed values; on the contrary, the smaller the value of R², the worse the fit of the regression line to the observed values.

Frequency distributions of *K_c_* are displayed in Figure 9 and the fitting correlation coefficients of grid 1, 2, 3 and 4 were 0.6102, 0.6069, 0.7951 and 0.6893, respectively. The varying ranges of *K_c_* of grid 1, 2, 3 and 4 were 5.36~15.55 GPa, 5.6~15.71 GPa, 6.43~18.44 GPa and 4.85~21.56 GPa, respectively. The mean fracture toughness $\bar{K_{c}}$ of four grids were 10.41, 11.23, 13.24 and 12.78 MPa·m^1/2^, respectively, and that of grid 3 was the largest. The mathematical expectations of the four grids were 10.08, 11.31, 13.24 and 12.78, respectively. The mathematical expectation of grid 2 and grid 4 was same as the mean value. The mathematical expectation of grid 1 was less than its average value, and a positive skewness distribution was presented, while that of grid 3 was greater than its average value, which presented a negative skewness distribution.

### 3.6. Relationships among Various Mechanical Properties

**Figure 10:**
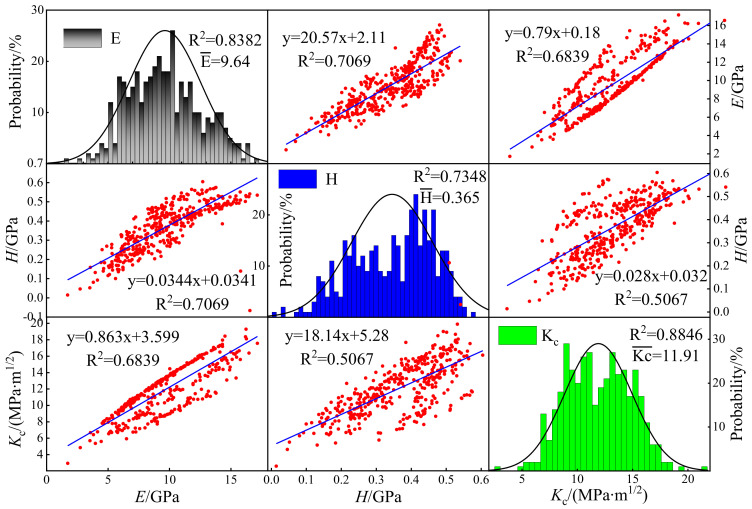
The correlation between *H* and *K_c_* was the lowest, and the correlation coefficient was 0.5067. In addition, the correlation coefficient between *E* and *K_c_* was 0.6839. It can be observed that data points present discrete distribution, which was probably caused by mineral heterogeneity, micro−cracks and micro−pores.

**Figure 11:**
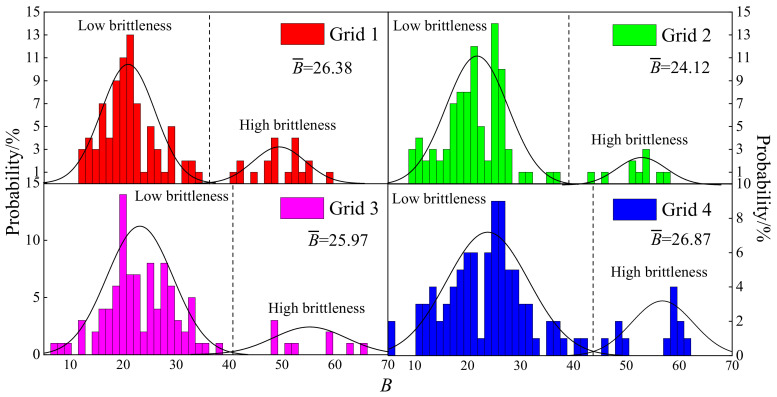
Frequency index *B*.

Figure 10 gives the relationships among various mechanical properties of the four grids after statistics, and the mechanical property distributions mostly followed the normal distribution. The frequency distributions of *E*, *H* and *K_c_* are displayed on the diagonal. The correlation coefficients between *E* and *K_c_* were higher than 0.8, which indicates a great fit. The correlation coefficient of *H* was a little worse with the value of 0.7348, but it was higher than that of the dispersed mesh. The mean values of *E*, *H* and *K_c_* were 9.64 GPa, 0.365 GPa and 11.91 MPa·m^1/2^, respectively. The correlations among the mechanical properties are demonstrated in Figure 11. 

## 4. Discussions

### 4.1. Evaluation of Micro-Brittleness Index

The micro-brittleness of the four grids can be obtained based on the Equation (16), and it reflects the friability of the indenter. By analyzing the brittleness index of numerous indenters, as displayed in Figure 11, the varying ranges of the brittleness index of the four grids were 11.31~58.67, 9.16~57.07, 7.46~65.69 and 10.52~61.32, respectively. Additionally, the mean micro-brittleness index $\bar{B}$ of the four grids was 26.38, 24.12, 25.97 and 26.87, respectively. The friability of grid 4 was the best based on the above analysis on the brittleness index. However, the analysis was invalid due to it ignoring the integrity and heterogeneity of the shale. Hence, a deep analysis is necessary in next section.

By investigating the distributions of the brittleness index in the four grids, it can be seen that the distribution of the brittleness index presented an obvious bimodal distribution, which could be divided into a low brittleness region and a high brittleness region. The boundary value of the low and high brittleness region in the four grids were 38, 40, 40 and 45, respectively. The mean brittleness indices of the low brittleness regions were 20.96, 21.05, 23.09 and 23.6, respectively. The mean brittleness indices of the high brittleness regions were 49.47, 51.77, 55.08 and 55.61, respectively. Additionally, the proportions of the high brittleness index were 19%, 10%, 9% and 14%, respectively. It can be concluded that the mean value of the brittleness index was small in grid 1, while the proportion of the high brittleness index was the largest, indicating that the friability of grid 1 was positive and higher than the other grids.

### 4.2. Distribution of Micro-Brittleness Index

The micro-brittleness index of a single point was obtained by pressing in and out of the nanoindentation head. In Section 4.1, the related statistical analysis was performed on the micro-brittleness index of a single point and the evaluation of the friability was finished. However, shale is strong heterogeneity material, and it is difficult to represent the overall characteristics of shale only through the analysis of a single point. Even if the mesh statistics are the same, the micro-brittleness will be different due to the various mesh distribution. In this paper, the indenter was moved along a serpentine path of the array by a selected starting point. By giving the array length and spacing between adjacent indentations, the brittleness index was matched with the spatial position of the indentation. This spatial distribution provides a way to determine the micro-brittleness distribution on the shale surface.

**Figure 12:**
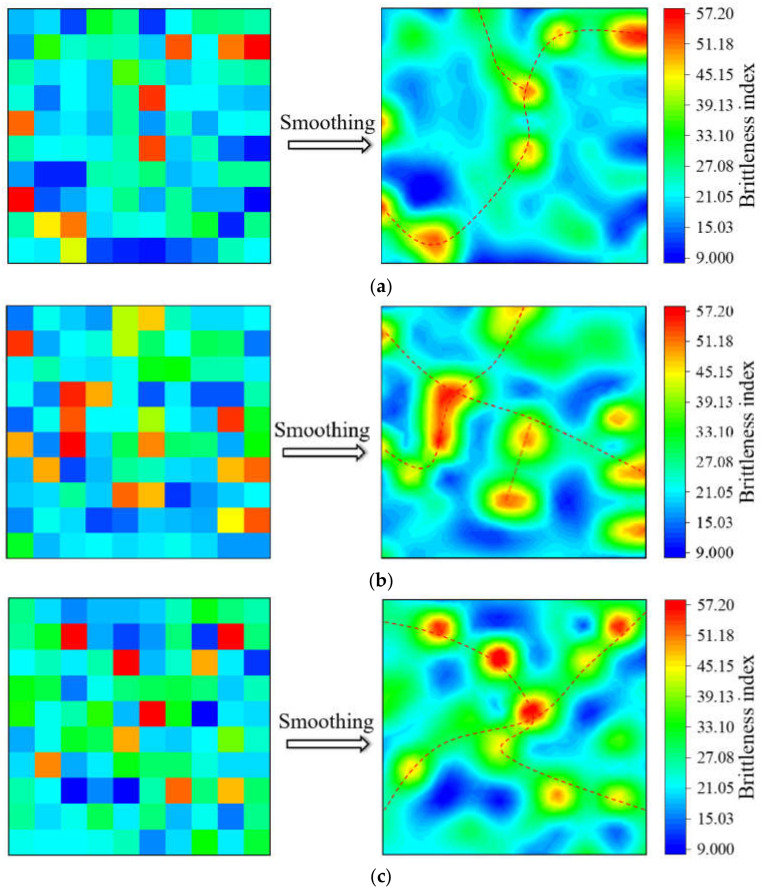

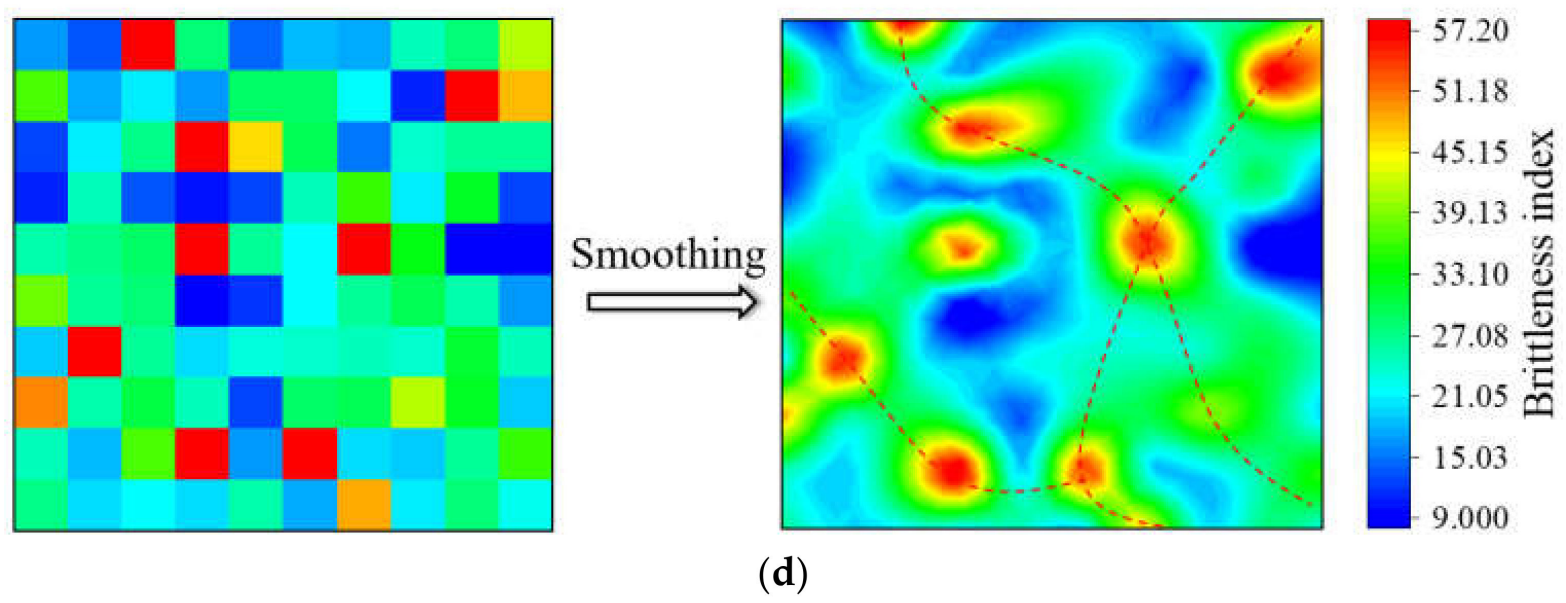
Friability surface distribution: (**a**) nanoindentation grid 1, (**b**) nanoindentation grid 2, (**c**) nanoindentation grid 3, (**d**) nanoindentation grid 4. The red dashed lines in Figure 12 are connections between highly brittle regions that form crack propagation channels.

Figure 12 shows the reference origin software and the method used was the difference method. As shown in the left of Figure 12, the brittleness index was drawn as a hotspot map according to its position, and the strong heterogeneity of the shale was observed. The color of the grid represents the level of the brittleness index. The red color represents a high brittleness index, which is scattered in the indentation grid, just as quartz grains are scattered in shale. The existence of high brittleness indentation points improves the overall friability. However, it is difficult to transfer the crack to the next point when two high brittle indentation points are far away from each other. The cloud diagram of the brittleness index distribution was obtained with step fuzzy processing in the right of Figure 12. When the crack extends to this point, it must extend to the high brittleness area. The high brittleness zones are connected to each other to form fracture propagation channels.

The content of the high brittleness indentation points was 19% in grid 1, which was higher than the other three indentation points. However, the concentration of the high brittleness indentation points led to a small number of crack expansion channels. When the crack expanded to grid 1, it was extremely easy to be absorbed by the low brittleness zone, resulting in the ability loss of the crack extension. Therefore, the high content of high brittleness in grid 1 was unvaluable for friability. In grid 3, there were a few high brittleness indentation points (9%), but these high brittleness indentation points were scattered and connected with each other to form multiple channels, which were conducive to the propagation of cracks. The brittleness index distribution of the four grids was analyzed. There were two channels for grid 1, four channels for grid 2 and grid 3 and five channels for grid 4. Therefore, grid 4 had the best friability based on the distributions of the brittleness index.

### 4.3. The Relationship between Micro-Brittleness Index and Mineral Distribution

**Figure 13:**
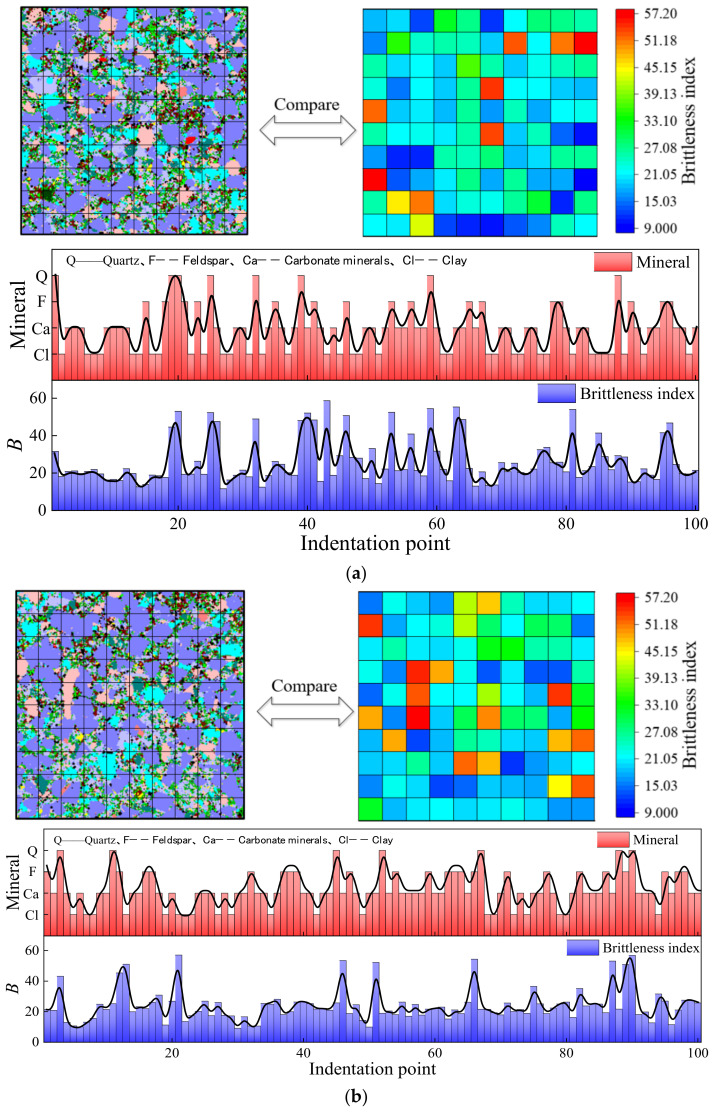

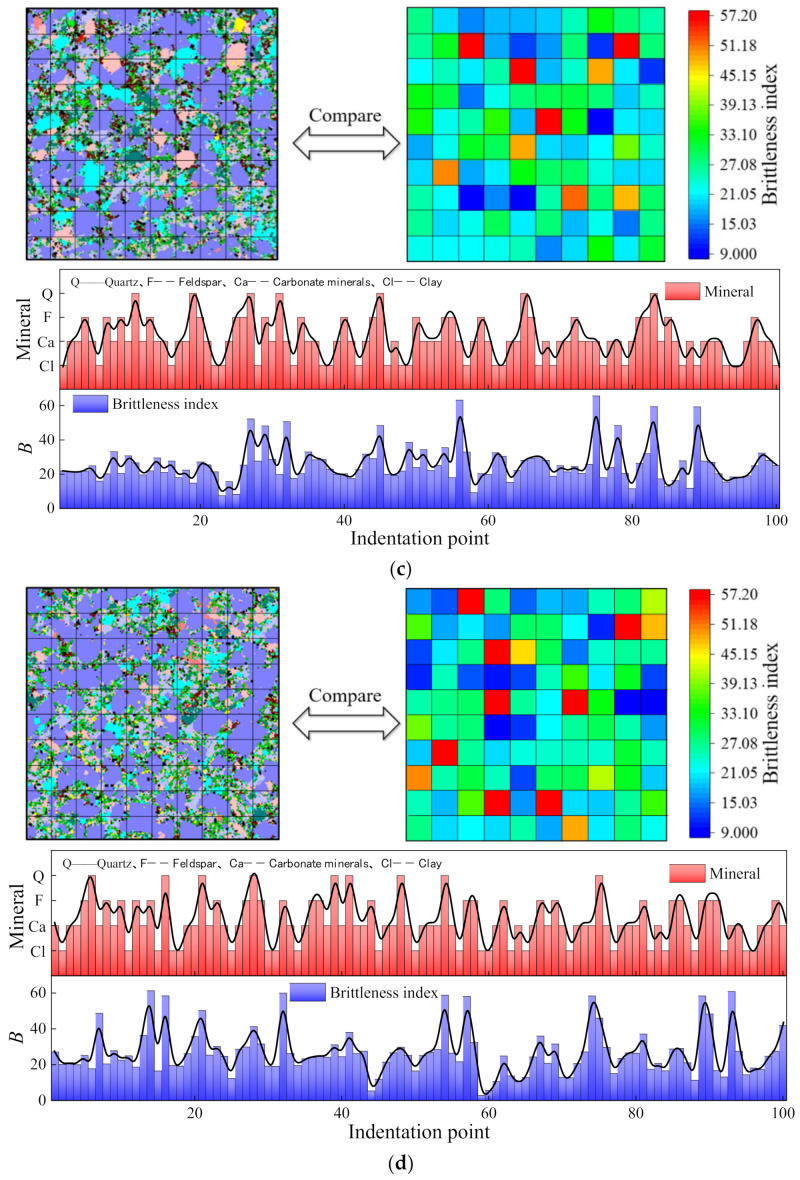
The relationship between brittleness index and minerals: (**a**) nanoindentation grid 1, (**b**) nanoindentation grid 2, (**c**) nanoindentation grid 3, (**d**) nanoindentation grid 4.

In previous studies, the same mineral was considered to have specific mechanical properties. However, different mineral cementations have a strong influence on their mechanical properties. Nanoindentation tests were carried out by the grid indentation method, and an indentation point was placed in a grid with various mineral connections. The categories of the main minerals in the grid were compared with the brittleness index obtained by indentation tests. Figure 13 shows the corresponding relationship between the minerals and the brittleness index. In grid 1, the quartz content, feldspar content, carbonate mineral content and clay mineral content was 14.09%, 13.79%, 48.16% and 17.43%, respectively. The indentation grid of the quartz-dominated grid accounted for 19%, basically corresponding to the high brittleness region. However, the brittleness index of the grid points by the feldspar-dominated and carbonate- dominated grids was in the middle. The grid points by the clay-dominated grid had a low brittleness index, but they also had a high brittleness index owing to the existence of brittle particles in the clay minerals. In grid 2, the quartz content was 9.52%, which corresponded to the high brittleness. The content of feldspar, carbonate minerals and clay minerals was 14.38%, 18.53% and 50.72%, respectively. In grid 3, the content of quartz, feldspar, carbonate minerals and clay minerals was 12.49%, 16.31%, 47.26% and 15.86%, respectively. The content of high brittleness was 9%, which was less than the quartz, and this was due to the low brittleness of some of the quartz surrounded by clay. In grid 4, the content of the quartz, feldspar, carbonate minerals and clay minerals was 8.43%, 17.3%, 51.52% and 15.37%, respectively. The proportion of high brittleness was 14%, which was much higher than that of quartz. This was because quartz particles are small and most of them were cemented with carbonate minerals, were part of the carbonate minerals and showed high hardness.

It can be seen that quartz played a very important role in the evaluation of microscopic brittleness. Quartz is highly brittle, and when it is cemented with other minerals, the overall brittleness is improved. Quartz takes on different shapes, most of which are round shapes. These quartz particles are relatively large and are formed during the diagenesis process. They co-deposited with carbonate minerals and cemented with clay minerals to form shale. In addition, many clusters of quartz can be observed. These small particles of quartz are produced in clay minerals. They may not be formed by diagenesis, but by the precipitation of siliceous material during the transformation of clay to form quartz. This is why some clay minerals also exhibit a high brittleness.

## 5. Conclusions

In this paper, nanoindentation technology was used to study the micro-mechanical properties of shale. Meanwhile, the indentation deformation was introduced through dimensional analysis and then the micro-brittleness index was proposed. Compared with the traditional dimensional method, the nanoindentation method was dimensionless, which could well characterize the physical meaning. The mineral composition, pore structure characteristics and surface mineral distribution of shale were characterized by XRD, SEM and QEMSCAN. The four regions of shale were compared in an analysis to explore the characteristics and distributions of shale micro-brittleness. The main conclusions are as follows:

(1) The micro-brittleness index of the shale ranged from 7.46 to 65.69 and the average brittleness index was 25.837. The distribution of the brittleness index presented an obvious bimodal distribution. The shale was divided into low brittleness and high brittleness around 40. It had a positive effect on the overall friability of shale although the proportion of these high brittle minerals was only about 15%.
(2) The brittleness distribution of the shale surface was obtained via the grid indentation method, and the distribution of the brittleness index presented a strong heterogeneity. The indentation points with a high brittleness index were scattered in the indentation grid. When the two highly brittle indentation points were closer, they were connected to each other to form a channel for crack propagation. When the content of the high brittle indentation points was high but too concentrated, it was not conducive to crack propagation.
(3) The distribution of the minerals obtained by QEMSCAN was meshed and then compared with the indentation grid to obtain the corresponding relationships between the brittleness index and minerals. The pattern of most indentation points was quartz with a high brittleness and clay with a low brittleness. However, there existed a variety of situations due to the various cementations between the minerals. When quartz was cemented with clay minerals, the brittleness decreased. When a small amount of quartz was cemented with carbonate, the indentation point showed a higher brittleness index. At the same time, the clay region also presented a high brittleness due to the presence of many small brittle particles in the clay minerals.

## Figures and Tables

**Table 1:** Mineralogical composition of shale sample.

Mineral Composition	Content/%
Quartz	12.5
K-Feldspar	3.0
Plagioclase	21.9
Calcite	23.2
Dolomite	25.9
Pyrite	1.4
Illite	7.34
Kaolinite	0.3
Chlorite	0.68
Kaolinite–montmorillonite	3.78

## Data Availability

All the data is available within the manuscript.
